# Attitudes Toward the Ethics of Research Using Social Media: A Systematic Review

**DOI:** 10.2196/jmir.7082

**Published:** 2017-06-06

**Authors:** Su Golder, Shahd Ahmed, Gill Norman, Andrew Booth

**Affiliations:** ^1^ Department of Health Sciences University of York York United Kingdom; ^2^ School of Nursing, Midwifery & Social Work University of Manchester Manchester United Kingdom; ^3^ School of Health and Related Research (ScHARR) University of Sheffield Sheffield United Kingdom

**Keywords:** review literature as topic, social media, ethics, research design, qualitative research

## Abstract

**Background:**

Although primarily used for social networking and often used for social support and dissemination, data on social media platforms are increasingly being used to facilitate research. However, the ethical challenges in conducting social media research remain of great concern. Although much debated in the literature, it is the views of the public that are most pertinent to inform future practice.

**Objective:**

The aim of our study was to ascertain attitudes on the ethical considerations of using social media as a data source for research as expressed by social media users and researchers.

**Methods:**

A systematic review was conducted, wherein 16 databases and 2 Internet search engines were searched in addition to handsearching, reference checking, citation searching, and contacting authors and experts. Studies that conducted any qualitative methods to collect data on attitudes on the ethical implications of research using social media were included. Quality assessment was conducted using the quality of reporting tool (QuaRT) and findings analyzed using inductive thematic synthesis.

**Results:**

In total, 17 studies met the inclusion criteria. Attitudes varied from overly positive with people expressing the views about the essential nature of such research for the public good, to very concerned with views that social media research should not happen. Underlying reasons for this variation related to issues such as the purpose and quality of the research, the researcher affiliation, and the potential harms. The methods used to conduct the research were also important. Many respondents were positive about social media research while adding caveats such as the need for informed consent or use restricted to public platforms only.

**Conclusions:**

Many conflicting issues contribute to the complexity of good ethical practice in social media research. However, this should not deter researchers from conducting social media research. Each Internet research project requires an individual assessment of its own ethical issues. Guidelines on ethical conduct should be based on current evidence and standardized to avoid discrepancies between, and duplication across, different institutions, taking into consideration different jurisdictions.

## Introduction

### Background

Social media are any Web-based computer-mediated tools to cocreate, share or exchange information, ideas, pictures or videos in virtual communities and networks (such as message boards, social networks, patient forums, Twitter, blogs, and Facebook) [1]. The availability of social media opens up new avenues for researchers to easily collect data, especially from sources that may have previously been difficult to access. This has led to a massive surge in social media analytics (whereby posts or chats are analyzed via qualitative methods or aggregate numerical data collection). The order of magnitude of data and the speed with which it is made available (approaching real time) make social media a potential tool to revolutionize health research [2]. Health-related social media analytics has taken many forms, including drug or product surveillance, monitoring disease or health patterns, or views or experiences of patients. Examples are pharmacovigilance (such as the discovery of adverse events), monitoring outbreaks of the flu epidemic, illicit drug usage or suicide patterns, and views on vaccinations and health service quality [3-29].

However, these new research avenues are not without ethical challenges [30-34]. In common with other research, potentially difficult considerations surround the purpose and value of the research, benefits and harm to participants, as well as privacy, informed consent, and confidentiality. However, Internet research is very different from traditional research and as such brings about many different ethical challenges. Whereas procedures are well established for obtaining ethical approval for traditional research, how far these can be transferred directly to Internet-mediated research is difficult to decipher. Whereas the ethical issues of social media research have been much debated [34-37], the attitudes of social media users (either posters or lurkers) and researchers have rarely been sought [38-42]. Researchers currently seek guidance from a wide variety of sources, such as individual institutions, research supervisors, subject specialist guidance [43], and increasing guidelines proposed specifically for research using social media [39,44-49].

This systematic review summarizes the existing research that has evaluated attitudes on the ethical considerations of research using social media. This will help to contribute to, and consolidate, current research practice as well as to clarify those ethical issues most pertinent to the public and researchers. This, in turn, will help guideline developers to formulate evidence-based guidelines for researchers conducting research using social media.

### Objective

This study aimed to systematically review the research evidence that has evaluated attitudes of social media users, researchers, and other stakeholders on the ethical considerations of using social media as a data source for research.

## Methods

### Inclusion Criteria

Due to the anticipated dearth of studies specific to health-related research and the potential for generalizability of other research, any research area was considered. To reflect the qualitative or mixed methods nature of the research, we adopted SPIDER (sample, phenomenon of interest, design, evaluation, research type) for defining the inclusion criteria [50]:

S- Sample: Any sample of people (such as social media posters or lurkers, researchers, academics or other stakeholders). No minimum sample size was implemented.

P- Phenomenon of interest: Attitudes held on the ethical implications of conducting research using social media analytics.

D- Design: Any qualitative data collection methods (eg, surveys, questionnaires, interviews, observations, or focus groups) independent of the analysis conducted. Discussion papers were excluded.

E- Evaluation: Any information on attitudes to the ethical implications of research using social media. Such information may be the primary or secondary focus of the study.

R- Research type: Qualitative (such as interviews or focus groups), quantitative (such as surveys or questionnaires with fixed responses only) or mixed methods (such as research which collates a combination of fixed and open-ended responses).

No date, language or publication type restrictions were applied to the inclusion criteria. However, financial and logistical restraints did not enable translation from all languages.

### Exclusion Criteria

Papers that are non–research based such as discussion papers were excluded. Such papers have been summarized elsewhere [35].

Research on the ethics of individual “look-ups” were excluded, for example, employers seeking information on employees or prospective employees, parents viewing their children’s posts, and health professionals seeking information on patients (or vice versa). Research on individual privacy or security issues such as fraud, cyberbullying, grooming, and child protection were also excluded.

### Search Methods

A total of 16 databases and 2 Internet search engines were searched in addition to handsearching journals and conferences (Multimedia Appendix 1). Databases were carefully selected to reflect the multidisciplinary nature of the review. Other methods included reference checking all included articles and any related systematic reviews, citation searching of key papers on Google Scholar and Web of Science, and contacting authors and experts.

### Search Strategies

The search strategies contained 3 facets—social media, ethics, and qualitative research. A date restriction of 1996 onwards was placed on the searches as blogging first began in 1997. No language limits were placed on the searches. The Embase search strategy is contained in Multimedia Appendix 2 and was translated as appropriate for each database.

### Selection of Studies

The results of the searches were entered into an EndNote library and duplicates were removed. The titles and abstracts were screened by 2 researchers independently (SG and GN). The full text of any potentially relevant articles was assessed for eligibility by 2 researchers independently (SG and GN or SA). Disagreements were resolved by consensus based discussion and, if necessary, a third reviewer.

### Data Extraction

Information was collected on the research question, the numbers of respondents, the characteristics of the participants (such as age and gender), the methods used to ascertain attitudes (such as interviews and survey), sampling methods or survey distribution (such as email and snowballing), questions or methods used to ascertain ethical considerations, and key findings. Data were extracted independently by 2 reviewers (SG and SA). Any disagreements were resolved by discussion or a third reviewer where necessary.

### Assessment of Methodological Quality

Two reviewers (SG, SA) conducted independent quality assessment using the methodological assessment tool—quality of reporting tool (QuaRT). This involves checking the reporting quality of the articles using 4 elements: (1) the question and study design, (2) recruitment and selection, (3) methods of data collection, and (4) analysis. Studies were categorized as “adequately reported” when a “yes” had been assigned against 2 or more criteria or “inadequately reported” where a study was assigned a single “yes” response, or no yes responses [51]. Any disagreements were resolved by discussion or a third reviewer where necessary.

### Analysis

Although the quality of the reporting of the included papers was assessed, no quality threshold was implemented. All studies which provided insight or contributed to the analysis were included.

We chose an inductive theme analysis with descriptive analysis rather than a framework approach. We acknowledge that a framework approach is an equally valid approach. However, we did not identify any framework completely compatible with our intended purpose. For instance, the most relevant framework identified was restricted to Twitter [52]. In addition, framework analysis has a risk of suppressing “interpretative creativity” and thus reducing some of the “vividness of insight” [53].

Inductive thematic synthesis aims to identify salient themes via coding of the data without the use of a preexisting coding frame, or any preconceptions held by the analysts [54] *.* We undertook coding in QSR NVivo Pro 11 by assigning text on a line-by-line basis to nodes developed by one author (SG) and then checked by a second author (SA). An aggregation approach to the synthesis of the data was applied with data from each study extracted and grouped together to form themes with supporting quotations. Finally, interrelationships between themes were assessed and organized into a structure to produce synthesized findings.

### Reporting

The enhancing transparency in reporting the synthesis of qualitative research (ENTREQ) statement of 21 items was used to report the stages of this review [55].

## Results

### Included Studies

**Figure 1:**
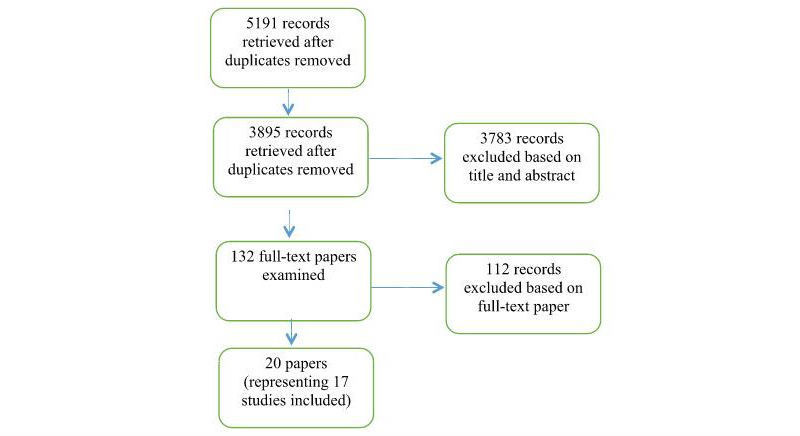
Flow diagram.

Of the 3340 records (4636 before duplicates removed) identified by the original searches in February 2016 and a further 555 unique records by update searches in July or August 2016, 132 full-text papers were retrieved of which 112 were excluded. Excluded studies were mostly concerned with personal privacy or security (such as bank details), or with individual “look-ups” (such as seeking information on a particular person; Multimedia Appendix 3). Overall, 17 studies (from 20 publications) met the inclusion criteria (Figure 1).

### Characteristics of Included Studies

Of the 17 studies, 12 explored the ethical concerns of social media users [38,40,56-67] and 5 asked researchers (mainly academics) [42,43,68-71] (Table 1 and Multimedia Appendix 4). The earliest study was published in 2001 and the latest in 2016, with 12 studies published after 2012.

Numbers of study participants were reported in 16 of the 17 studies. The smallest study involved 26 respondents and the largest study 2260. The total number of participants was over 5453.

In 7 studies the participants were from a single country: the United Kingdom (4), the United States (2), or Australia (1). The other studies either explicitly indicated an international participant coverage (3 studies) or implied an international coverage of participants (7 studies)—most of which used social media for recruitment or study observation.

8 studies used surveys of participants’ attitudes, whereas others carried out more in-depth research and analysis using interviews (6 studies), or focus groups (4 studies). A mixture of open and closed questions was used. Three studies used observational or experimental techniques; with an original design devised by the authors (4 studies used more than one technique).

The majority of the studies did not indicate the demographic details of the respondents; however, in those that did males and females were well represented. Some studies specifically targeted young people [38,59,65].

5 studies were specifically concerned with health-related research [38,57,64,66,69], whereas the remaining studies tended to be more general.

### Quality Assessment

Only 3 studies (from 4 reports) were assessed as “inadequately reported” [42,56,67,71] (Multimedia Appendix 5). The contribution to the synthesis of these 3 “inadequately reported” studies was assessed as being limited. These 3 studies did not impact on the presence of concepts within the synthesis and had only a marginal effect on the detail within the concepts.

### Analysis

Whereas some included studies reported summary data from surveys or questionnaires, the majority of studies presented author interpretations supported by verbatim extracts from participants.

### Emerging Themes

Some responses spanned multiple themes and attempted to categorize overall reactions to social media research use. Many closely related themes underpin the diverse attitudes exhibited by the study respondents. The framework we adopted from our emerging themes was: the 2 actors (researchers and social media users), managing their relationship (consent), and framing the context (responsibilities of the social media site (Tables 2 and Multimedia Appendix 6). Some studies assessed multiple themes in a more integrated and quantifiable way and we consider these first under general reactions.

Within the arena of “researchers,” we identified themes of the perceived benefit of the research, the affiliation or type of researcher, the validity of the methods used to conduct the research, and the risks to the researchers themselves. Those themes related to the “social media users” were concerned with the risks involved with particular concern for vulnerable groups. Linked to these risks were the intended purpose of the social media poster and their ability to self-regulate through personal censorship or privacy settings. The next theme related to “consent” and the importance of and difficulties of informed consent and research disclosure. The last theme was regarding “responsibilities”—either via the social media site (including the platform and site administrators) or legal requirements (Tables 2 and Multimedia Appendix 6).

### General Reactions

Three studies attempted to quantify general reactions to social media research. Moreno found that over half (56.1%, 74/132) of university students “strongly like” or “somewhat like” the concept of using Facebook for research by university researchers, nearly a third (28.8%, 38/132) were “neutral” and only 15.2% (20/132) were “unsure or uneasy” or had an “overt concern” [38].

Williams found that 37.2% (N=564) of social media users were “not at all concerned” with their social media information being used by university researchers, whereas 46.4% (N=564) were “slightly concerned,” 11.2% (N=564) “quite concerned,” and 5.2% (N=564) “very concerned” [67]. Evans found that 60% (N=1250) of social media users felt that “sharing individuals’ social media data with third parties, such as the government or companies, for research purposes” should not happen and 32% (N=1250) felt that “sharing overall numbers of social media data with third parties, such as the government or companies, for research purposes (but not linked to individuals)” should not happen [59].

Respondents in Evans may have been less positive because they were given use of examples relating to government and companies rather than university researchers [38,59,67]. The more positive attitudes in Moreno may be linked to surveying younger people [38,59,67].

### Researchers

#### Perceived Benefit of Research

Research was considered more acceptable if “it’s going to a good cause” [65] and was “morally right” or for “general good” (such as social benefit or to help others) [38,40,56-59,62-65]. Some respondents were more specific—stating that social media research could give a voice to patients and other groups, uncover true prevailing issues, and improve patient care [57,58,65];

I have no reservations about your mining information from forums...it will provide much information about the human side of illness and how individuals singly and collectively approach and cope through sharing. Dare I say its importance cannot be understated.

Diabetic forum user 57

I kind of think it’s cool when it’s stuff that’s like the flu, because then that’s how they know to get the vaccines to a place.

Twitter user 64

Others described the general benefit of social media research as a precondition to acceptance [38,40,57,65] or considered if the benefits outweighed the risks [40,64]. Others felt positive provided a caveat or set of conditions had been met, such as informed consent (see following sections).

Whereas some stipulated the good that should come from social media research, others stated the research they would not like to see, such as research with a “bad intention” [38,65], for commercial gain [40,57,62,63] or to drive an agenda [40].

The strong feeling for the “public good” meant that some felt service to the greater social good was more important than individual privacy concerns [64]. However, others considered that social media users’ desire for privacy should take precedence over researchers’ goals [66].

#### Type of Researcher

Linked to the purpose of the research was the affiliation of the researcher. The type of organization or company influenced whether or not respondents viewed research as “good quality” and user concerns about use of social media information [40,42,58,59,64,65,67,69]. Generally, respondents were less concerned about use of social media information by university researchers, than by students [58], the police, government organizations [67], commercial organizations [40,59,64,67] or journalists [58]. But no difference was reported between health organizations and researchers [69].

Social media users who preferred not-for-profit researchers (such as academics) to commercial organizations did so because the former were felt to be more “productive,” more “ethical,” and “not exploitative.” Furthermore, users did not like their social media posts being used to generate a profit for others [40,58,64].

**Table 1:** Brief summary of characteristics of included studies.

Source		Number of respondents	Characteristics of the participants	Survey distribution	Specific questions or methods used to ascertain ethical considerations	Key findings identified in terms of the ethical considerations raised
**Researchers as respondents**	Alim 2014 [43]	64	International and interdisciplinary researchers and academics	Emailed questionnaire	*Open and closed questions*	Researchers aware of ethical issues but require clarity in informed consent and public and private data.
	Bakardjieva 2001 [56]	NR^a^	Mailing list discussants	Mailing list discussion	Discussions provoked by post	An ethical approach to Web-based research is practically achievable.
	Carter 2015 [68]	30	Academic staff from UK university	Emailed Web-based survey	Respondents asked to agree, disagree or neither to 12 statements.	Recognize importance of avoiding deception and gaining consent, but acknowledge problems. Most disagreed that studying public social media data is same as studying documented text and disagreed that individuals wouldn’t be identified if anonymous.
	Denecke 2014 [69]	45	International Medical Informatics Association (IMIA) social media working group members	Mailing list members	3 questions asked	Different social media platforms should be managed in different ways in terms of confidentiality and privacy. Individuals should be deidentified and cited only indirectly.
	McKee 2009 [70]	30	International and interdisciplinary researchers from corporate research centers and academia.	Contacted known researchers	Used open-ended interviews	Researchers strived to follow “do no harm” principle. Agreement that there is no blanket approach to Internet research ethics.
	Woodfield 2013 [71]/Salmons 2013 [42]	465	International and interdisciplinary researchers	Program of Web-based and offline activities including conference and Twitter chat	Open discussions in which ethics consistently raised	Discussions focused on informed consent, confidentiality or anonymity, role and safety of the researcher, and research setting or social media platform. Concern of a lack of agreement.
**Social media users as respondents**	Beninger 2014 [40]	34	Adult male and females	British social attitudes 29 (BSA 29) survey and external recruitment agency	Focus groups and interviews using vignettes	Conditional acceptance of using social media for research.
	Bond 2013 [57]	26	Male and females	Diabetes forums	Web-based semistructured asynchronous (email) interviews	Agreed forum posts in the public domain and aggregated information could be used by researchers.
	Chen 2004 [58]	47	Mailing list owners and moderators or long standing members on sensitive and controversial topics	Emailed mailing lists and newsgroups	Survey questionnaire	Animosity toward researchers. Research should be conditional on research identify disclosure, informed consent, and feedback.
	Evans 2015 [59]	1265+	Aged 13 to 75 years and broadly reflective of UK population	NR	Web-based survey, workshops or interviews included example research projects	60% do not support use of social media data for research. Terms and conditions not sufficient for consent and need option to opt out. Biggest factor in likelihood to approve research is whether data is public.
	Hudson 2004 [60] and Hudson 2005 [61]	Up to 2260	Chatroom users on ICQ chat—range of topics including geographical region or language, age-orientated, romance or friends, adult or sexuality, technical, trivia avoided sensitive discussions such as “breast cancer survivors”	Monitored chatrooms	Recorded if “kicked-out” of chatroom and messages on why or comments to researchers	Kicked out of 63.3% of chatrooms when message posted about research. Reasons were— prohibition of spamming, opposition to being studied, general requests to leave, and insults.
	Michaelidou 2016a [62] and Michaelidou 2016b [63]	405	Adult male and females	NR	Focus groups and Web-based survey	10-item scale of transparency, legality, approval, privacy concerns, permission, vulnerability, reward, consumer responsibility, protection, and terms.
	Mikal 2016 [64]	26	Male and females with average age 26 years. Over half with depression history	Advertised on list serves, discussion boards, and local Internet community websites	Focus group s *emistructured* interviews	Relatively positive view provided data are anonymous and aggregated to protect identities.
	Monks 2015 [65]	48	13-14 years old Australian school students	Sample of convenience drawn from students participating in a leadership workshop	Focus groups with 3 main open questions	Some concerns about privacy but open to the use of social media for research if opportunity to provide consent and assured of confidentiality and anonymity.
	Moreno 2012 [38]	132	Male and female adolescents aged 18-19 years within US university	Used Facebook to identify students	At end of interview about health, asked “We identified participants for this study by looking at publicly available Facebook profiles. Do you have any thoughts about that?”	Endorsement by 26 respondents. 48 were “fine” with it, 38 were neutral or no specific comments, 12 were uneasy, 8 had overt concerns.
	Petersen 2013 [66]	27	Members of Medical Webmasters (MWM), an open, unmoderated list and Patient Advocates in Research (PAIR), a closed, unmoderated list for cancer patient advocates	Posted on 2 electronic lists	Survey	Two themes emerged. Respondents believed journalists should seek permission from list members and/or webmasters and viewed members’ desire for privacy as taking precedence over researchers’ goals.
	Williams 2015 [67]	564	Social media users	Web-based survey	Survey with open and closed questions	37% are not at all concerned about their social media information being used by university researchers, whereas 46% are slightly concerned, 11% are quite concerned, and 5% are very concerned.

^a^NR: not reported.

**Table 2:** Summary of coding framework.

Concept headings	Concepts derived for coding	Definitions
Researchers	Perceived benefit of research	Overall outcome or intention of the research to do “good.”
	Type of researcher	The affiliation of the researcher (such as university or commercial company). This is associated with the perceived benefit of the research.
	Validity of the research methods	High or low validity of the methodology used, including risk of bias.
	Risks to researchers	Any risks that the researcher is exposed to.
Social media users	Risks to social media users	Any risks that the social media users are exposed to either individually or as a group.
	Vulnerable groups	Groups could be determined as vulnerable by either their individual characteristics or the topic discussed.
	Original purpose of posts	The intent of the poster at the time the message was placed.
	Privacy and self-regulation	The public versus private nature of social media and the need for anonymity or confidentiality. Connected to this issue is self-regulation whereby individuals control content.
Consent	Informed consent	Permission for posts to be used in a study.
	Researcher disclosure	Researchers being transparent and honest about their intent. This can be either up-front or at a later stage.
Social media site responsibilities	Terms of service	Also known as “terms of use” or “terms and conditions,” these are the rules agreed to in order to use social media sites.
	Site administrators	Site administrators, list administrators or list moderators are often in charge of maintaining a discussion or mailing list.
	Legality	Refers to legal issues, regulation or government oversight and includes issues of copyright.
	Type of platform	The type of social media platform, for example closed or open, personal or professional. This is connected to issues of public versus private space, and the original purpose of the postings.

#### Validity of the Research Methods (High and Low)

Whereas users’ perceptions of the validity of social media research was partially influenced by the researcher affiliation, they also discussed its methodological rigor on its own merits. Attitudes were divided as to whether social media research could be viewed as high or low validity research, particularly compared with more traditional research methods. Those users who viewed social media research as high quality cited it as a means of quick access to vast amounts of timely information and large samples to mitigate the effect of false information or extreme views and improve research accuracy [40]. Anonymity of posts was also thought to encourage open and honest opinions and discussions particularly about sensitive issues or nonconventional or “politically incorrect” views. Research using social media was also seen to avoid biases inherent in having to answer questions in the presence of others, such as in a survey [40].

In contrast, other social media users were concerned about the low validity of the research in terms of quality of the data, representativeness, and poor methodological approach [40,58,64,65]. Concerns were raised about inaccurate or false data or accounts with people severely limiting what they post on the Web (see self-regulation), and behaving differently off and on the Web [40,58 64,65];

I’ve never once posted anything negative. So if you took that data, it would not be accurate, because of course I have had bad days or sad days.

Twitter user [ 64]

Social media posters were not considered representative of the general population and using social media would lead to “only the loudest voices heard” [40,64].

Some social media users were skeptical not only about the data posted on social media but also about the accuracy of the methods of using social media, and biased research;

You can’t even get targeted advertising right, what makes you think public health accuracy is going to be any better?

Twitter user [ 64]

Many researchers in the fields covered by this list do research solely to “prove” that our illness are faked or psychological. Most of us do not care to operate with people like that. Any truly unbiased research is fine.

Chemical-injury mailing list owner [58]

#### Risks to Researchers

Insults or being “flamed” were the most common threat posed to researchers [58,60,61]. Researchers spoke from experience of the need for care that they do not become victims to trolls and to separate their “researcher” persona from their “personal” persona and thus protect the boundaries between their professional and personal lives [60,61,71].

The potential for more extreme harm was cited in countries with governments which control Internet access and communications [70]. For example, an associate professor carrying out a study in Central Asia stated that local researchers could be risking their lives by conducting social media research;

I can’t get anyone to work with me right now because the Uzbek government just passed a law that anyone accused of giving sensitive information to foreigners will be accused of treason, and the law doesn’t define what is sensitive information. Now the penalty for treason in Uzbekistan is death, I believe.

Associate professor, University of Washington [70]

### Social Media Users

#### Risks to Social Media Users

Social media users were also worried about the risks of judgment or ridicule or unsolicited attention on the Web and, more seriously, “abuse” or bullying [40,43,64,65,70]. Other possible harm included exploitation from organizations or use by the police or courts for purposes of prosecution in divorce cases, child custody cases or lawsuits [40,58]. Other social media users felt very uneasy about social media research or felt it was “creepy” or “scary” [38,58,60,61,64,65]. Respondents commonly held the perception that posters are being “exploited” or “used,” with researchers using social media posters to “get someone to do their work for them” was widely held [58,65].

Risks were associated with data being taken out of context, used inappropriately or the poster being identifiable. Users were concerned at the potential to distort the context in which something was said or that findings would be used to defend or promote something that was not intended (see purpose and validity of the research) [40,64,65]. Some respondents, although happy for researchers to use verbatim quotes, felt that “if it actually involves taking your comments and interpreting it, then it’s a very different thing” [40]. Use of verbatim quotes, rather than some form of interpretation [65], was one solution to taking comments out of context. However, this bought about issues of anonymity and privacy.

Researchers were generally aware of the risks to social media users (even with anonymized data) and considered these risks in their studies [43,70]. In addition, researchers were worried that the risks were not taken seriously by international review boards (IRBs) [43,70]. Deidentification of social media posts was seen as crucial to minimize negative consequences [65]. However, using verbatim quotes could compromise individual anonymity (see privacy).

Whereas users were well aware of the risks, they exhibited a feeling of apathy [38,40,56,64] with risks just being something to be accepted and the only way to stop it happening being to stop using social media [38,40,56,64];

With some of the stuff I write, I am uncomfortable thinking it is going to be accessible for a long time but this is after all the Internet and it’s hardly private...The alternative, (that is) total privacy is to sit here in my house alone and not communicate. I’d give it about three weeks before total insanity set in.

Discussion list member [56]

Risks extended beyond individuals to social media groups. It was considered important to maintain social media as a protected space where members may speak openly without concern that their words will be shared outside the group [65,66]. Researchers were seen by some as an intrusion which can destroy the dynamics and enjoyment of using social media and curtail freedom of expression [58,65,66,70]. This was reiterated by some users who reluctantly self-regulated their posts [65]. Some users even felt that the damage to communication and community within these forums could lead to people not participating or sites closing [58,70].

#### Vulnerable Groups

Certain groups such as children and teenagers [42,43,62,63,65,71], individuals suffering from mental health issues [64], and even the deceased [66] were perceived as vulnerable and thus required extra emphasis on respect and caution to counteract this vulnerability [65,66]. The legal context and government practice of the country from which the post originates may also affect the potential for harm. For example, homosexuality is illegal, or at least taboo, in certain regions. Thus, individuals could be exposed to severe harm if their sexual orientation were publicized [70]. Risks to professional reputations and careers were also raised for those, such as school teachers or health professionals, with responsibility for potentially vulnerable or impressionable individuals [40].

Whereas some social media users thought ethical principles of research should be upheld regardless of the topic of the research [40,65], others thought that topics of a sensitive or personal nature needed more consideration [58-61,65,70]. The sensitive nature of some discussion groups was cited as good reason to prohibit or discourage researchers;

Our code of conduct explicitly prohibits information gathering from SPALS (subsequent pregnancy after a loss support) for other than immediate personal use. …Privacy and confidentiality are also concerns. We don’t want to attract the “research-paparazzi.”

List owner [58]

#### Original Purpose of Posts

When social media users post on social media, they may have no expectation that this would ever be used for research [40,56,57,65,71]. The intended audience may be limited to friends and family and possibly “friends-of-friends” [65,71]. Considerations of the wider implications and access by third parties are not likely to be at the forefront of many social media users’ minds [40,56,57,65].

Even if people are aware of the public nature of social media, people may still “get carried away with themselves when they are writing (on social media platforms)” [40] and then “once it is on there, to try and get rid of it, it’s too late or it’s too hard” [40]. Thus, there is a need to consider carefully the impact of reporting of verbatim social media data no matter how open or public a site is considered to be [71];

I was also very irritated with people who used that argument that we should not ask for informed consent because it is easy to get into the groups. It is the participants’ purpose for being in the group that is important in a way and their feelings about what kind of space this is.

Researcher, Norwegian University of Science and Technology [ 70]

#### Privacy and Self-Regulation

Whether social media should be seen as a public or private space occasioned contention as well as confusion [40,42,43,56-66,68-71] and was the principal factor in the likelihood of approval for social media research projects [59]. Some social media users believe that “there is no such thing as privacy online” [40], so once information is posted it is available to the public and thus can be accessed and used for research purposes [38,40,57].

Despite the commonly held view that social media are public, some privacy was still expected and this raises caution for Internet research [38,56,57,62-64]. Some users still expressed discomfort about it being used—although appreciated how contradictory this may appear [57,63];

I write a blog about my experience of diabetes and would feel very aggrieved if I found any of it quoted in a medical research paper without having been asked. I realise this is slightly contrary (since I am posting and effectively actively encouraging readership) but nevertheless it would feel like “theft” of my content.

Diabetic forum user [ 57]

An expectation of privacy resulted, in part, from a lack of understanding of the extent of the public nature of social media exchanges [56]. Navigating the privacy settings of social media isn’t always simple or straightforward. Some social media users did not know what is public or private on social media or what their settings were [38,64,65]. Some users did not understand the permanent nature of social media data, how extensive data reach can be, and the big data computational tools that can be used to analyze posts [64]. Users also felt it was easy to forget or not think about this while posting (see risks) [57].

Confidentiality and anonymity were thought to help protect privacy [42,43,70,71]. Social media users emphasized the importance of anonymity [40,59,64,65,67,69] with approximately three-quarters preferring to remain anonymous [59,67]. Users were also more likely to accept research if it looked at deauthored data or used overall aggregated numbers [40,57-59,64,65,69].

Some respondents (both users and researchers) identified the challenge of using quotations and maintaining anonymity [43,57,68,70] given that quotes, even if anonymized, can be traced back to their origins using a Google search [57,70]. These views were confirmed in a small Web-based survey of academics from a single UK university where only 10 out of 30 researchers agreed it was “very unlikely that individuals will be able to be identified if social media datasets are anonymised” [68].

One solution to using direct quotes was to cite only indirectly or to paraphrase quotes [69]. This, however, could have implications for those social media users’ who were concerned about their posts being taken out of context (see risks).

Respondents who disagreed with the need for anonymity believed that users are responsible for managing their identity as people “can always be anonymous if you want to be” [40]. For example, users could use a username unrelated to their real name, utilize privacy settings, and select what to share on the Web [40,43,62-65,68]. The idea that there is no such thing as privacy was reiterated (52) and as such self-regulation was key [64];

I think our generation is gravitating towards (the idea that) privacy is not to be expected anymore. You have to create it yourself. You have to enable it yourself, because it just doesn’t exist anymore.

Twitter user [64]

This idea that self-regulation should be relied upon was reflected in the results of the survey of UK academics where 17 out of 30 agreed “it is the responsibility of individuals to rethink how they use social media if they are unwilling for their online public behavior to be studied by researchers” [68].

These views were inextricable from views on informed consent as social media users who actively “self-regulated” did not think researchers needed to gain consent [40].

### Consent

#### Informed Consent

Generally social media users were divided as to whether they agreed or disagreed that social media research required informed consent from posters [67]. Users and researchers who did not feel informed consent was necessary tended to feel that informed consent was implied by the public nature of social media [40,43,57,64]. Others felt that anonymization removed the need for informed consent [40,43,57,64,65,69].

Other social media users expressed reservations feeling that researchers should obtain permission for use [57,58,62,63,65,66]. They linked this to the original intention of the post [57], data ownership, or to difficulties of anonymizing direct quotes [57,65]. Attitudes appear to be changing as users learn from experience and social media develops;

I have allowed this in the past, but I feel that they should get permission first.

Mailing list owner [ 58]

Gaining consent was seen as part of common decency and not solely to ensure good ethical practice [40,57,65]. Researchers were felt to have a moral responsibility toward Web-based content [40,57,65] and to protect citizens from violations linked to social media research [62,63].

Some researchers tended to assume that proceeding without informed consent was acceptable because social media are public [38,40,43,57,68].

Only publicly visible data was extracted so we thought that, because the data was publicly available, no ethics applied.

Researcher [ 43]

This was reiterated in the survey of 30 UK academics where 10 felt that there is no need for informed consent if social media were publically accessible [68].

However, they also raised the issue of whether posts represent a “human subject” or text [58,70].

If I think of Perry’s comments as the letter for the editors, I don’t have to get any informed consent, but if I think of it as a personal conversation, I have to get informed consent.

Doctoral student, York University in Canada [ 70]

This issue was not discussed among social media users but when asked, 22 out of 30 UK academics disagreed that “studying public data on social media is essentially same as studying documented text” [68].

Some users saw the necessity for informed consent as depending upon both the content and type of analysis. Many thought that sensitive, personal posts or posts with a “sexual, political or religious” focus required informed consent [40,58] and were more accepting of the use of aggregate data, generalizations or observational overviews than case studies or the use of quotes (this was interconnected with anonymity) [56-58,64,69,70].

Both social media users [40,65] and researchers [43,68,70] felt that it was difficult to implement informed consent. Challenges related to the large amounts of data involved, the impossibility of getting informed consent from all the users, and difficulties in how and whom to ask [43,70]. The impracticalities of detecting minors were also highlighted [43,65]. However, where respondents favored consent, they did not think that logistical burdens offered a justification for not seeking permission [40,65].

#### Research Disclosure

Many social media users felt that the collection, access, and use of social networking data should be transparent [40,56-58,60-66]. Some felt that authors of postings should know how their comments might be used “up-front” at the time of producing them [40,42,56] with an option to opt-out of research (or even better, an opt-in) [58-61];

Any researcher that joins a mailing list should identify themselves as such as soon as they have joined-opt better yet before they have joined and ask permission of the list owner. As a person I have a right to know I am being experimented on or studied.

List owner [ 58]

Many researchers agreed that being explicit and transparent as possible about one’s role as a researcher was the best possible action [68,70] whereas some were uncertain about when it is appropriate to collect data without disclosing their identities [71]. Deception of social media users was generally seen as unacceptable [68].

Respondents drew a distinction between naturally occurring social media data and data stimulated by a researcher’s intervention. It was more important for a researcher to make themselves and their intentions known in advance when participating in forums [71].

### Social Media Site Responsibilities

#### Terms of Service

Most researchers factor in consideration of “terms of service” for the social media platforms from which they extract data into their research planning [43]. Whereas the vast majority of social media users were aware of “terms of service,” neither researchers nor most social media users agreed that this is sufficient for informed consent [59,64,67,68]. Terms of service were considered too long, dense, and confusing [40,64]; “constantly changing” [40]; and unread by most members of social media sites [40,64,65,67,69,71]. A few social media users felt that the public openness and accessibility of the platform of social media (such as Twitter or LinkedIn) implies that third parties may use the data [40]. Thus, researchers should not rely upon the terms of service.

#### Site Administrators

There was an absence of consensus over the role of site administrators. Some social media users thought that researchers need to gain permission from the list owner [58,66,69], in addition to the users’ permission;

No individual or entity should be using it (private forum) for research without explicit permission from both the people who writes the message as well as the people/group who runs the mailing list.

List owner [ 58]

Others were vehemently opposed to list owners giving permission on behalf of members;

I think it would be a complete betrayal if (admin) were to give permission on behalf of the members.

Diabetic forum user [57]

#### Legality

Some social media users thought government oversight or regulation should ensure the ethical use of data and protect the rights of users [64]. Some suggested a law against collecting information about social media users [62,63] and not gaining consent was compared with “hacking” [40]. Others suggested that governmental oversight was unnecessary leading to fears of Orwellian monitoring [64].

The public nature of social media platforms and their content raises the issue of data ownership [40,57,62,63,66,70]. Some social media users thought that users automatically surrender their right to ownership by posting and that as they are public they are “uncopyrighted so they are ‘free’ for anyone to use” [56] or the social media platform owns all data on the site [40,57,64].

However, other social media users and researchers thought that users own the intellectual property of content they post and that posts should be treated in line with copyright laws [40,43,56,58,59,66,69,70]. This requires that proper referencing or acknowledgment is in place [40,57,58,70];

If someone decided to republish my post in another forum or document, I would expect my comments to be kept in context and credited to me.

Diabetic forum user [ 57]

However, including a “handle” or the Web-based username in a reference was perceived as problematic by some social media users who valued anonymity over credit [40,57,58,70].

#### Type of Platform

The type of social media website, such as open and closed groups or sites with privacy settings, also influenced whether consent was considered implicit [40,64,65,69,71];

I feel if a forum is viewable to the public, IE you don’t have to be a member to view any of the forum threads, then you or anyone else can use any of the information you find on any forum.

Diabetic forum user [ 57]

Twitter and Web-based open forums were seen as inherently public forums [40,64,65]. In contrast, Facebook has explicit privacy control settings and was therefore viewed differently [40,64];

It all comes down to the fact that we know that we’re using Twitter and it’s public. I think I might honestly feel differently about that if it were Facebook, because I do feel like there is some degree of privacy in Facebook.

Twitter user [ 64]

iMessage and other messaging functions were viewed as private spaces with closed conversations [58,65];

A mailing list unlike a sidewalk has a membership list and only members are part of that list… the mailing list retains an identity as a PRIVATE forum. With that in mind, no individual or entity should be using it for research without explicit permission..

List owner [ 58]

Social media websites with a fun, social purpose are likely to contain much more “personal” content and were therefore viewed differently from websites with a professional aim such as LinkedIn [40].

Users were also more concerned about researchers accessing and using photos than written content because text could have been written by anyone—whereas it is more difficult to falsify photos [40].

## Discussion

### Principal Findings

This systematic review demonstrates the need to understand a complex array of interrelated and challenging factors with respect to ethical considerations in social media research. Diverse important issues and concerns remain to be addressed, but consensus proves difficult to achieve. Respondent views varied considerably from complete “animosity” to being “overly positive.” In between were attitudes of conditional acceptance (for instance social media research only to be undertaken if informed consent is gained or the site is public) and complete apathy. In addition, even views from the same respondent were conflicting with many being well aware of their contradictory views. The comprehensiveness of this review enables us to develop a broader view of the contrasting issues rather than being restricted to findings from a single study or single population that is not as transferable.

The differing attitudes can partially be explained by the heterogeneity of respondents, their different understandings of social media research, and different methodologies used in each included study. In particular, Twitter users and the younger generation tended to be more accepting of social media research. Research using social media is a relatively new phenomenon. Some respondents had little understanding of social media research particularly in the earlier studies or were unaware of the range of examples. Social media users were more likely than researchers to evaluate the perceived benefit of the research and the validity of the research when considering the acceptance of social media research. Researchers, on the other hand, were also more likely than social media users to be concerned about risks to researchers themselves.

There was general agreement on several issues. Whereas there was strong support for social media research that is for the “good” of society (and thus greater acceptance of university researchers)—this should not offer researchers carte blanche or an opportunity to ignore ethical principles. There are different definitions of “beneficial” research and some people could still be at risk of harm. Respondents acknowledged possible risks with these being more complex for such groups as, children, adolescents or vulnerable adults [38,72-75] or for discussions on sensitive topics. Risks to social media group dynamics and freedom of expression have been highlighted in the literature [36,76] and this review demonstrates the strength of feeling about the importance of keeping safe and supportive environments for people to post on. The potential benefits, therefore, should still be weighed up against any potential harm. However, often researchers considered general principles such as “do no harm” as difficult to follow [70].

Respondents were much more likely to support the use of numerical aggregate data (such as overall statistics) than qualitative research involving quotes or interpretation of quotes. This view was almost unanimous [40]. Respondents agreed overwhelmingly that the terms of service of social media platforms are infrequently read and should not be relied upon. This finding may be related to newsworthy cases where terms of service were relied upon. Over time much has been learnt through trial and error. As a consequence, platforms such as Twitter and Facebook have changed their terms of service with much tighter control over external research [76,77]. However, this review does not suggest that users feel this is the best way forward.

Respondents also thought that the conduct of social media research should not just obey the principles of the law (although there was little agreement as to what the law is) but also follow ethical principles and a moral obligation.

Many issues encountered little agreement and conflicting attitudes. The boundaries between public availability and privacy were particularly complex. The public nature of social media has been used to support current practice, whereby a minority of studies apply for institutional ethics board or other approval and most studies do not mention ethical approval [43,73,78-80]. Social media research, therefore, often involves a lack of informed or valid consent with group members often unaware that they are being monitored [76,80,81]. Whereas some social media users seemed happy with this lack of consent, quoting the public nature of social media and the potential to self-regulate, others strongly opposed this on the grounds of privacy, original intent of the post, risks to users, and ownership of the data. These arguments are similarly rehearsed in the literature [34,43,78,82-86].

Rooted within the concepts of risk to users are the issues of privacy and traceability or anonymity of the poster [34,38,39,41,48,71-73,77,81,87-93]. Whereas some respondents were happy for use of anonymous posts, others wanted to be cited (as is the case for published works). Both users and researchers displayed a lack of understanding of the difficulty in upholding anonymity [80]. In 2006, deanonymization of social media users was carried out by journalists of the New York Times [94], and in 2011 data aggregated and allegedly anonymized by researchers from Harvard were deanonymized [83,95]. Covert participation inside Web-based communities has also occasioned controversy [96].

Respondents lacked clarity with regard to informed consent, when it should be implemented, and how [42,71]. However, users agreed that it is unacceptable to use names and direct quotes without consent and that it is practically difficult to obtain informed consent. The issue of informed consent was highlighted in 2012 when Facebook manipulated users’ posts in an experiment to influence people’s mood. This caused much anger and dismay among users who were unaware they were part of any experiment [97,98]. The included studies in this review, however, did not examine the ethics of manipulation of sites and this needs separate consideration.

Researchers may access content posted on the Web without interacting with the individuals who wrote the posts or even considering them “human subjects.” The “human subject” or “published author” debate, however, was little discussed in the included studies, yet, has important implications for ethical approval and is often debated in the literature [70].

The absence of an overarching consensus with regard to social media research ethics is apparent. Many complex ethical dilemmas persist [73]. Whereas researchers have found it useful to read and understand ethical considerations faced by other researchers, they find it challenging to translate approaches used in another research context to their own particular research [70]. Each research project, therefore, requires individual consideration of its ethical issues. Just as a blanket approach to the ethics of traditional research (such as surveys or focus groups) would prove unsuitable so, too, we should resist a “one rule fits all” to social media research. Researchers should consider the type of research (such as aggregate, qualitative); the nature of their topic (whether sensitive or trivial); as well as issues of anonymization, confidentiality, informed consent, privacy, and the benefits and risks involved. The ability to undertake Internet research may depend on the level of trust and confidence the public have in the research being undertaken when people choose to contribute to studies or make posts publically available [77]. Koene [77] considers a possible revolt with a call for a public backlash and boycott of Internet-mediated research. This review does not suggest that social media users feel as strongly as Koene [77] suggests, but we should still be mindful of these issues.

### Limitations

Although the heterogeneity of included studies contributes strength to this review, this also makes it challenging to arrive at definitive conclusions. In particular, studies differed in their methodological approaches and by their presentation of results.

Social media are constantly evolving and have changed considerably since the first of the included studies was undertaken in 2001. It would be interesting to uncover how people’s perception of the ethical issues in social media research have changed; however, unfortunately the sparsity and heterogeneity of studies made it challenging to reveal any time trends.

Whereas additional non-database searches (such as citation searching and reference checking) were used in an attempt to overcome the difficulties in searching for this type of research, it is likely that some relevant studies remain unidentified. The review was also limited to papers in English or for which a translation could easily be obtained.

### Conclusions

It remains unlikely that a consensus on the ethical considerations on using social media research will ever be reached. Each Internet research project requires an individual assessment of its ethical issues and selection of the most appropriate methodological approach.

Whereas the issues raised in this review suggest that ethical considerations in using social media for research are complex and require thoughtful consideration, this should not deter researchers from conducting social media research. Contributions from social media offer a more immediate time window than experiences documented in formally published qualitative research. This is because social media data can be analyzed immediately whereas published qualitative findings may take at least two years from data collection, through peer review and the wider editorial process, to publication. To a large degree such contributions may be unfiltered and unfettered and less subject to the influence of the researcher. They are less constrained by the temporal and spatial limitations encountered when planning and conducting qualitative research. However, social media contributions are public offerings largely written in the knowledge that they could be read by a wider audience. They offer a perspective that is often stripped, or at the very least, lacking a grounding in context and that may challenge representation and interpretation. Thus, we cannot afford to miss the considerable potential of social media research and its unique contribution to knowledge.

Guidelines for ethical conduct should be based on the available best practices and standardized to avoid discrepancies and duplication from one institution to another. This methodological review is offered to initiate ongoing discussion within the research community of how such guidelines might be formulated. It highlights the importance of properly conducted social media research of benefit to the public. It also highlights the need to consider informed consent and privacy and researchers should not rely solely on regulation but have a moral and ethical duty to consider social media users and the main purpose of social media groups.

## Appendix

Multimedia Appendix 1 Sources searched.

Multimedia Appendix 2 Embase search strategy.

Multimedia Appendix 3 Excluded studies.

Multimedia Appendix 4 Characteristics of included studies.

Multimedia Appendix 5 Quality of reporting in included studies.

Multimedia Appendix 6 Results matrix for studies by emerging themes.
