# Supplementary material for: Attitudes Toward the Ethics of Research Using Social Media: A Systematic Review
Source: J Med Internet Res. 2017 Jun 6;19(6):e195. doi: 10.2196/jmir.7082 (PMC5478799; doi:10.2196/jmir.7082)
Supplement: Multimedia Appendix 1 [file jmir_v19i6e195_app1.pdf]

Appendix 1: Sources Searched

The sources searched are listed below;

Databases

ACM Digital Library
ASSIA
CINAHL
Conference Proceedings Citation Index – Science
Conference Proceedings Citation Index – Social Science & Humanities
Embase
Google
Google Scholar
Health Management Information Consortium (HMIC)
INSPEC Direct
LISTA (Library, Information Science & Technology Abstracts)
MEDLINE (including PubMed)
OpenGrey
Proquest Dissertations & Theses: UK & Ireland
PsycINFO
Science Citation Index (SCI)
Social Science Citation Index (SSCI)
Zetoc

Handsearching of Journals

Journal of Medical Internet Research

Handsearching of Conference Proceedings

International AAAI Conference on Weblogs and Social Media (ICWSM)
Ethics and Social Media Research (Conference)
