# Supplementary material for: Attitudes Toward the Ethics of Research Using Social Media: A Systematic Review
Source: J Med Internet Res. 2017 Jun 6;19(6):e195. doi: 10.2196/jmir.7082 (PMC5478799; doi:10.2196/jmir.7082)
Supplement: Multimedia Appendix 2 [file jmir_v19i6e195_app2.pdf]

Appendix 2: Embase Search Strategy

Database: Embase <1974 to 2016 January 27>
Search Strategy:
----
1 social media.ti,ab,kw. (3664)
2 social medias.ti,ab,kw. (31)
3 web 2*.ti,ab,kw. (1566)
4 blog*.ti,ab,kw. (1522)
5 wiki.ti,ab,kw. (495)
6 wikis.ti,ab,kw. (174)
7 weblog*.ti,ab,kw. (107)
8 web log*.ti,ab,kw. (69)
9 facebook.ti,ab,kw. (1960)
10 face book.ti,ab,kw. (24)
11 tweet.ti,ab,kw. (189)
12 tweets.ti,ab,kw. (374)
13 tweeting.ti,ab,kw. (73)
14 twitter*.ti,ab,kw. (1320)
15 you tube.ti,ab,kw. (46)
16 youtube.ti,ab,kw. (867)
17 social web.ti,ab,kw. (48)
18 social software.ti,ab,kw. (30)
19 social medium.ti,ab,kw. (17)
20 crowd sourcing.ti,ab,kw. (98)
21 crowdsourcing.ti,ab,kw. (287)
22 instant messaging.ti,ab,kw. (175)
23 microblogging.ti,ab,kw. (76)
24 social bookmark*.ti,ab,kw. (47)
25 patientslikeme.ti,ab,kw. (74)
26 patient forum*.ti,ab,kw. (54)
27 discussion forum*.ti,ab,kw. (704)
28 online forum*.ti,ab,kw. (302)
29 on line forum*.ti,ab,kw. (21)
30 chat forum*.ti,ab,kw. (19)
31 chatforum*.ti,ab,kw. (1)
32 chatroom*.ti,ab,kw. (68)
33 chat room*.ti,ab,kw. (317)
34 (online adj2 discussion*).ti,ab,kw. (551)
35 (on line adj2 discussion*).ti,ab,kw. (137)
36 discussion board*.ti,ab,kw. (281)
37 online chat*.ti,ab,kw. (107)
38 on line chat*.ti,ab,tw. (4)
39 (online adj2 communication*).ti,ab,kw. (378)
40 (on line adj2 communication*).ti,ab,kw. (38)
41 digital media.ti,ab,kw. (300)
42 digital medias.ti,ab,kw. (1)
43 personal health message*.ti,ab,kw. (3)
44 user comment*.ti,ab,kw. (54)
45 patient* posts.ti,ab,kw. (3)
46 user* posts.ti,ab,kw. (7)
47 internet accounts.ti,ab,kw. (6)
48 internet sites.ti,ab,kw. (610)
49 message board*.ti,ab,kw. (165)
50 web scale.ti,ab,kw. (16)
51 google plus.ti,ab,kw. (10)
52 user generated.ti,ab,kw. (225)
53 consumer generated.ti,ab,kw. (22)
54 online health content.ti,ab,kw. (8)
55 on line health content.ti,ab,kw. (0)
56 internet narrative*.ti,ab,kw. (9)
57 social network* site*.ti,ab,kw. (696)
58 online social network*.ti,ab,kw. (356)
59 on line social network*.ti,ab,kw. (8)

60 social networking.ti,ab,kw. (1800)
61 on line comment*.ti,ab,kw. (3)
62 online comment*.ti,ab,kw. (56)
63 internet forum*.ti,ab,kw. (178)
64 web forum*.ti,ab,kw. (54)
65 internet media.ti,ab,kw. (41)
66 web media.ti,ab,kw. (5)
67 sentiment analysis.ti,ab,kw. (66)
68 Social Media/ (5191)
69 Crowdsourcing/ (363)
70 or/1-69 (16003)
71 exp ethics/ (231165)
72 (ethics or ethic or ethical).ti,ab,kw. (121995)
73 (moral adj2 (policy or principle*)).ti,ab,kw. (469)
74 informed consent.ti,ab,kw. (50944)
75 anonymity.ti,ab,kw. (2108)
76 anonymous.ti,ab,kw. (22303)
77 privacy.ti,ab,kw. (11829)
78 confidential*.ti,ab. (16531)
79 intellectual property.ti,ab,kw. (2292)
80 Confidentiality/ (23824)
81 Privacy/ (11178)
82 morality/ (28708)
83 or/71-82 (355738)
84 70 and 83 (1735)
85 interview$.ti,ab. (309675)
86 qualitative.ti,ab. (173080)
87 experience$.ti,ab. (1052070)
88 themes.ti,ab. (44561)
89 questionnaire*.ti,ab. (486945)
90 ethnograph*.ti,ab. (7656)
91 fieldwork.ti,ab. (2415)
92 field work.ti,ab. (1158)
93 key informant.ti,ab. (2385)
94 focus group*.ti,ab. (32525)
95 (("semi-structured" or semistructured or unstructured or informal or "in-depth" or indepth or "face-to-face" or structured or guide) adj3 discussion*).ti,ab. (2977)
96 qualitative analysis/ (39429)
97 qualitative research/ (34163)
98 exp interview/ (181127)
99 exp questionnaire/ (474801)
100 survey$.ti,ab. (561744)
101 discourse analysis/ (638)
102 (discourse adj2 (analysis or stud*)).ti,ab. (1393)
103 observational study/ (83934)
104 exp observational method/ (2441)
105 (observational adj2 (analysis or stud*)).ti,ab. (104134)
106 or/85-105 (2391541)
107 84 and 106 (795)
