# Supplementary material for: Attitudes Toward the Ethics of Research Using Social Media: A Systematic Review
Source: J Med Internet Res. 2017 Jun 6;19(6):e195. doi: 10.2196/jmir.7082 (PMC5478799; doi:10.2196/jmir.7082)
Supplement: Multimedia Appendix 3 [file jmir_v19i6e195_app3.pdf]

Appendix 3: Excluded Studies

Reference	Reason for exclusion
Adams SA, van Veghel D, Dekker L. Developing a Research Agenda on Ethical Issues Related to Using Social Media in Healthcare Lessons from the First Dutch Twitter Heart Operation. Cambridge Quarterly of Healthcare Ethics. 2015;24(3):293-302.	Social media for educational use not for research.
Aguirre E, Mahr D, Grewal D, et al. Unraveling the Personalization Paradox: The Effect of Information Collection and Trust-Building Strategies on Online Advertisement Effectiveness. Journal of Retailing 2015;**91**(1):34-49.	States that a firm's strategy for collecting information from social media websites is a crucial determinant of how customers react to online personalized advertising. No further data.
Aimeur E; Lawani O, Dalkir K. When changing the look of privacy policies affects user trust: An experimental study. Computers in Human Behavior 2016;58:368-379	Personal privacy policies and trust.
Almadhoun N, Dominic P, Lai Fong W. Perceived security, privacy, and trust concerns within Social Networking Sites: The role of Information sharing and relationships development in the Malaysian Higher Education Institutions' marketing. Piscataway, NJ: IEEE, 2011:426-31.	Personal privacy concerns. Examines security, privacy, and trust in SNSs.
Amos C, Zhang LX, Pentina I. Investigating Privacy Perception and Behavior on Weibo. Journal of Organizational and End User Computing. 2014;26(4):43-56.	Personal privacy from Government intrusion in China.
Antheunis ML, Tates K, Nieboer TE. Patients' and health professionals' use of social media in health care: motives, barriers and expectations. Patient education and counseling 2013;**92**(3):426-31.	Privacy issues cited as potential barrier to social media use. Likely personal privacy.
Barnes SB. Issues of attribution and identification in online social research. In: Johns MD, Chen, S. S. and Hall, G. J. (Eds.), editor. Online Social Research: Methods, Issues, and Ethics. New York: Peter Lang; 2004. p. 203-22.	Posted message asking if members considered their conversations to be public or private. Several members thought messages should be private. No further data.
Bassett EH, O'Riordan K. Ethics of Internet research: Contesting the human subjects research model. Ethics and Information Technology. 2002;4(233-47).	Discussion with case study of research on named website. States that users behaviour suggests they viewed it as spatial and 'parochial'. Not enough detail.
Bateman PJ, Pike JC; Butler BS. To disclose or not: publicness in social networking sites. Information Technology & People 2011;24(1):78-100.	Personal privacy. Correlation of perceived privacy and self-disclosure on social media.
Battles H. Exploring Ethical and Methodological Issues in Internet-Based Research with Adolescents. International Journal of Qualitative Methods. 2010;9(1):27-39.	Discussion of ethical concerns in light of a study examining discussions on human papillomavirus (HPV) vaccine on Internet message board.
Bedi R, Gove NR, Wadhai VM, et al. Hippocratic Social	Questions regarding third party use and concern about how

Network. 2013 Fifth International Conference on Computational Aspects of Social Networks 2013:50-55.	social networking sites using information but limited to personal information. Defines personal information as the information which can be used to identify the user in the network (e.g. profile picture, email id, mobile number. address .friend list or other networks etc.).
Benson V, Saridakis G, Tennakoon H. Information disclosure of social media users: does control over personal information, user awareness and security notices matter? Information Technology & People 2015;**28**(3):426-41.	Investigates the link between information disclosure, control over personal information, user awareness and security notices with online companies.
Bevan-Dye AL, Akpojivi U. South African Generation Y students' self-disclosure on Facebook. South African Journal of Psychology 2016;46(1):114-129.	Personal data exposure and social media sites.
boyd AW. A longitudinal study of social media privacy behavior. Dissertation Abstracts International: Section B: The Sciences and Engineering 2012;**72**(10-B):6099.	Disclosure of personal information over time and trust of social media sites.
boyd D, Crawford K. Critical Questions for big data. Information, Communication & Society. 2012;15(5):662-79.	Discussion paper.
boyd D, Marwick A. Social Privacy in Networked Publics: Teens' Attitudes, Practices, and Strategies. Paper to be presented at Oxford Internet Institute's "A Decade in Internet Time: Symposium on the Dynamics of the Internet and Society" on September 22 2011	Explores teenagers views on personal privacy and social media. Concerns about parents viewing sites. No mention of researchers.
Brito KD, Garcia VC, Durao FA, Meira SRD. How People Care about Their Personal Data Released on Social Media. 2013 Eleventh Annual International Conference on Privacy, Security and Trust (Pst). 2013:111-8.	Personal data only. Not enough data.
Brady ME. An analysis of blogger motivations and approaches to privacy. University of Essex, 2012	Examines privacy issues relating to blogging about others.
Chai S. Three essays on behavioral aspects of information systems: Issues of information assurance and online privacy. Dissertation Abstracts International Section A: Humanities and Social Sciences. 2009;70(5-A):1706.	Personal privacy and online security only.
Chen HL, Beaudoin CE, Hong T. Protecting Oneself Online: The Effects of Negative Privacy Experiences on Privacy Protective Behaviors. Journalism & Mass Communication Quarterly 2016;93(2):409-429.	Personal privacy and online security only.
Chen J, Ping JW, Xu YJ, Tan BCY. Information Privacy Concern About Peer Disclosure in Online Social Networks. Ieee Transactions on Engineering Management 2015;62(3):311-324.	Personal privacy only.
Child JT, Pearson JC, Petronio S. Blogging, Communication, and Privacy Management: Development of the Blogging	Examines privacy rules employed by college students on their blogs to regulate privacy.

Privacy Management Measure. Journal of the American Society for Information Science and Technology 2009;**60**(10):2079-94.	
Chiu CJ, Menacho L, Young SD. The Association Between Age and Ethics-Related Issues in Using Social Media for HIV Prevention in Peru. Ethics & Behavior. 2016;26(2):99-109 11p.	Survey responses from 221 adult men on their experiences and perceptions of ethical issues relating to a HIV prevention study on social media. Not specifically research using social media.
Cirucci AM. Redefining privacy and anonymity through social networking affordances. First Monday 2015;20(7):1-9.	Discussion paper.
Clemons EK, Wilson JS. Family Preferences Concerning Online Privacy, Data Mining, and Targeted Ads: Regulatory Implications. Journal of Management Information Systems 2015;32(2):40-70.	Reports on targeted advertising and price discrimination based on users personal details and behavior.
Colbert J, Lehmann E, Agarwal P, et al. Crowdsourcing the diagnosis: Results of a national survey of patient attitudes towards physicians sharing deidentified patient images online. Journal of General Internal Medicine 2015;**30**((Lehmann E.) Cornell University, Ithaca, United States):S130-S31.	Participants were asked if they would give permission to have their picture posted online.
Comabella CCI, Wanat M. The use of social media to recruit participants in cancer care research: Challenges and opportunities. Psycho-Oncology 2013;**22**:163.	Conference abstract. No data.
Conway M, O'Connor D. Social Media, Big Data, and Mental Health: Current Advances and Ethical Implications. Current opinion in psychology 2016;9:77-82.	Summarises social media-based applications of NLP.
Conway M. Ethical issues in using Twitter for public health surveillance and research: developing a taxonomy of ethical concepts from the research literature. J Med Internet Res 2014;16:e290.	Systematic review of the literature on ethics of using Twitter for research.
Cooner TS, Knowles, A, Stout B. Creating a Mobile App to Teach Ethical Social Media Practices. Social Work Education 2016;35(3):245-259.	Evaluation of an app to teach about ethical use of social media.
Cosgrove V, Grimm D, Gliddon E, Lauder S, Dodd S, Berk M, Suppes T. Ethical dilemmas of participant safety monitoring in online clinical research. Bipolar Disorders 2015;17:140.	Conference poster citing ethical dilemmas of a self-help program for bipolar disorder.
Crick MR. Power and Surveillance in YouTube's Digital Neighborhood: A Case Study of College Students in the Bronx. Dissertation Abstracts International Section A: Humanities and Social Sciences 2012;**73**(5-A):1609.	Personal privacy concerns, such as email and financial information. Companies selling information.
Custers B, van der Hof S, Schermer B. Privacy Expectations of Social Media Users: The Role of Informed Consent in Privacy Policies. Policy & Internet 2014;**6**(3):268-95.	Personal privacy concerns and expectations.
Davison C, Speed E, Gunell C. Understanding patient and professional conceptions of "trust". Lessons from the PEBL project, an experiment in anonymous micro-blogging for the	Micro-blogging project as a method for public/patient feedback to NHS service providers and commissioners.

continuous assessment of patient experience. International Journal of Integrated Care. 2013;13:2.	
Dawson P. Our anonymous online research participants are not always anonymous: Is this a problem? British Journal of Educational Technology. 2014;45(3):428-37.	Study on the prevalence of direct quotes leading to identification.
Eysenbach G, Till JE. Ethical issues in qualitative research on Internet communities. BMJ. 2001;323:1103-5.	Discussion paper. Reports on a study on Dejanews archive of views on research surveys. States responses often negative but no further details.
Eysenbach G, Wyatt J. Using the Internet for surveys and health research. J Med Internet Res. 2002;4(2):E13.	Discussion paper
Feng Y, Xie W. Teens' concern for privacy when using social networking sites: An analysis of socialization agents and relationships with privacy-protecting behaviors. Computers in Human Behavior 2014;**33**:153-62.	Personal privacy concerns. Exams the privacy-protecting behaviours of teens and the role of their parents and SNS usage.
Fisher J, Clayton M. Who gives a tweet: assessing patients' interest in the use of social media for health care. Worldviews on Evidence-Based Nursing. 2012;9(2):100-8.	Healthcare not health research.
Fuchs C. studiVZ: social networking in the surveillance society. Ethics and Information Technology 2010;**12**(2):171-85.	Awareness of privacy policies and third party sharing of personal information.
Gehner M, Oughton D. Ethical challenges in social media engagement and research: considerations for code of engagement practices. Journal of Radiological Protection 2016;36(2):S187-S192.	Discussion paper.
Georgalou, M. 'I make the rules on my Wall': Privacy and identity management practices on Facebook. Discourse & Communication 2016;10(1):40-64.	Explores self-presentation on Facebook.
Ginnis S, Evans H, Barlett J, et al. Unlocking the value of social media work package ethics 2015	Literature review
Grajales F, Clifford D, Loupos P, Okun S, Quattrone S, Simon M, et al. Social Networking Sites and the Continuously Learning Health System: A Survey. Washington, DC: Institute of Medicine; 2014 https://nam.edu/wp-content/uploads/2015/06/VSRT-PatientDataSharing.pdf.	Reports on 2 web-based surveys on the acceptability of sharing personal health data with doctors and researchers and to help other patients. States that 91% of PLM believed their health data shared on social media were being used to improve care but most patients (52%) did not share their data outside PLM. No other information specifically referring to social media.
Gustafson DL, Woodworth CF. Methodological and ethical issues in research using social media: a metamethod of Human Papillomavirus vaccine studies. BMC Medical Research Methodology. 2014;414(127).	Systematic review
Hall GJ, Frederick D, Johns MD. NEED HELP ASAP!!!": A feminist communitarian approach to online research ethics. 2004) Online Social Research: Methods, Issues, and Ethics, Peter Lang, New York. 2004:239-52.	Presents examples from public from student research project using social media.

Haynes D. Social media and risk. (cover story). CILIP Update. 2014:30-2.	Reports on the risks of using social media as perceived by library and information staff.
Hays R, Daker-White G. The care.data consensus? A qualitative analysis of opinions expressed on Twitter. BMC public health. 2015;15:838.	Uses Twitter to identify opinions on care.data data (whereby patients' medical records are regularly uploaded to a central database).
Henderson M, Johnson NF, Auld G. Silences of ethical practice: Dilemmas for researchers using social media. Educational Research and Evaluation. 2013;19(6):546-60.	Discussion paper.
Hermann F, Janssen D, Schipke D, et al. Acceptance of Future Technologies Using Personal Data: A Focus Group with Young Internet Users. Human-Computer Interaction, Pt I 2009;**5610**:431-37.	Asked “Are you feeling watched when surfing the net?” and “Do you think it’s good if companies use your data (e.g. which sites you’re visiting) to give you personalized offers and advertisements?” Refers to personal information.
Herron M. Ethical issues in undertaking internet research of user-generated content: a review of the literature. 2011.	Systematic review
Horvath K, Danilenko G, Williams M, Simoni J, Amico K, Oakes J, et al. Technology Use and Reasons to Participate in Social Networking Health Websites among People Living with HIV in the US. AIDS & Behavior. 2012;16(4):900-10.	Survey of use of social media. Personal privacy as a barrier only.
Hu C, Zhao L, Huang J. Achieving self-congruency? Examining why individuals reconstruct their virtual identity in communities of interest established within social network platforms. Computers in Human Behavior 2015;**50**:465-75.	Examines why individuals reconstruct their identity in social network communities.
Humphreys L, Wilken R. Social media, small businesses, and the control of information. Information, Communication & Society 2015;**18**(3):295-309.	Explores how companies use social media.
Im EO, Chee W. An online forum as a qualitative research method: practical issues. Nursing research 2006;**55**(4):267-73.	Describes the practical issues encountered in implementing an online forum as a qualitative component of a larger study on cancer pain experience.
Jamal A, Coughlan J, Kamal M. Mining social network data for personalisation and privacy concerns: a case study of Facebook's Beacon. International Journal of Business Information Systems 2013;**13**(2):173-98.	Case study with blog data on issues with personal identity rich information (e.g. name, email, location, telephone numbers, etc.) of customers for marketing purposes but not research purposes.
Johns MD, Hall GJ, Crowell TL. Surviving the IRB review: institutional guidelines and research strategies. In Online social research: methods, issues and ethics. Edited by Johns MD, Chen S-LS, Hall GJ. 2004. 105-24. Peter Lang: New York.	Survey of researchers experiences with IRB applications.
Kim H, Giacomin J, Macredie R. A Qualitative Study of Stakeholders' Perspectives on the Social Network Service Environment. International Journal of Human-Computer Interaction 2014;**30**(12):965-76.	Study on personal privacy and security issues in using social media and impact on design of software agents.
King SA. Researching Internet Communities: Proposed Ethical Guidelines for the Reporting of Results. The Information	Gives only one example of a posters reaction to finding out their notes were the subject of research. No qualitative research

Society 1996;**12**(2):119-28.	methods.
Krasnova H, Spiekermann S, Koroleva K, et al. Online social networks: why we disclose. Journal of Information Technology 2010;**25**(2):109-25.	Study on what motivates users to disclose personal information. Not related to social media research.
Krasnova H, Veltri NF, Ieee. Privacy Calculus on Social Networking Sites: Explorative Evidence from Germany and USA. 43rd Hawaii International Conference on Systems Sciences Vols 1-5 2010:3039-48.	Study on how cultural differences impact self-disclosure on social media.
Lee SSJ, Vernez SL, Ormond KE, Granovetter M. Attitudes towards social networking and sharing behaviors among consumers of direct-to-consumer personal genomics. Journal of Personalized Medicine. 2013;3(4):275-87.	Study on attitudes of consumers disclosing share personal genetic information. Includes disclosure on social media but not researchers using the information from social media.
Li J. A Privacy Preservation Model for Health-Related Social Networking Sites. Journal of Medical Internet Research 2015;17(7):1-1.	Discussion paper and model on personal privacy and security only.
Liu S, B. The Emerging Ethics of Studying Social Media Use with a Heritage Twist. “Revisiting Research Ethics in the Facebook Era: Challenges in Emerging CSCW Research.” Workshop at ACM Conference on Computer-Supported Cooperative Work (CSCW 2010), Savannah, GA 2010.	States that “few participants who chose to remain anonymous and requested that their content not be reused publicly.” But no data.
Liu ZL, Min QF, Zhai QG. Smyth, R. Self-disclosure in Chinese micro-blogging: A social exchange theory perspective. Information & Management 2016;53(1):53-63.	Personal privacy and its relationship with self-disclosure.
Lunnay B, Borlagdan J, McNaughton D, Ward P. Ethical use of social media to facilitate qualitative research. Qualitative Health Research. 2015;25(1):99-109.	Used Facebook to facilitate in-depth explorative photo elicitation research. Discusses ethical implications of researchers seeking to communicate with participants on social media. Quotes from participants on public aspects of social media and befriending researchers. No further data.
Martin N, Rice J, Martin R. Expectations of privacy and trust: examining the views of IT professionals. Behaviour & Information Technology. 2016;6.	Personal privacy.
McKee R. Ethical issues in using social media for health and health care research. Health policy (Amsterdam, Netherlands). 2013;110(2-3):298-301.	Discussion paper.
Moreno MA, Fost NF, Christakis DA. Research Ethics in the MySpace Era. Pediatrics. 2008;121(1).	Discussion paper
Moreno MA, Goniu N, Moreno PS, Diekema D. Ethics of social media research: common concerns and practical considerations. Cyberpsychology, behavior and social networking. 2013;16(9):708-13.	Discussion paper
Nemec Zlatolas L, Welzer T, Hericko M, et al. Privacy antecedents for SNS self-disclosure: The case of Facebook.	Study on how privacy issues determine self-disclosure. Not issues on research.

Computers in Human Behavior 2015;45:158-67.	
Nicholas A, Bailey JV, Stevenson F, Murray E. The Sexunzipped trial: young people's views of participating in an online randomized controlled trial. Journal of Medical Internet Research. 2013;15(12):e276.	Study to determine participants' views of the acceptability and validity of the online trial methodology used in the pilot RCT. Not using social media posts for research.
Ofcom. Internet use and attitudes: 2013. Metrics Bulletin Ofcom: London 2013.	Social media usage statistics and statistics on terms and conditions read but no relevant data.
Padrez KA, Ungar L, Schwartz HA, Smith R, Hill S, Antanavicius T, Brown DM, Crutchley P, Asch D, Merchant RM. Linking social media and medical record data: a study of adults presenting to an academic, urban emergency department. BMJ Quality & Safety 2016;25(6):414-423.	Patients asked if they would be willing to participate in a study about social media and health. Reasons given. No further questioning or details obtained.
Paine C, Reips U-D, Stieger S, et al. Internet users' perceptions of 'privacy concerns' and 'privacy actions.'. International Journal of Human-Computer Studies 2007;**65**(6):526-36.	Personal privacy of issues such as viruses, Spam, Spyware, Hackers, Access to personal information, security, identity theft, Trojan and deception/honesty.
Quinn K. An ecological approach to privacy: "Doing" online privacy at midlife. Journal of Broadcasting & Electronic Media 2014;**58**(4):562-80.	Mostly examines personal privacy although states that "participants also expressed concern about potential misuse of their information by such third parties". But no data.
Quinn, K. Why We Share: A Uses and Gratifications Approach to Privacy Regulation in Social Media Use. Journal of Broadcasting & Electronic Media 2016;60(1):61-86.	Personal privacy such as employers, university and police monitoring or looking up individuals.
Reddick CG, Chatfield AT, Jaramillo PA. Public opinion on National Security Agency surveillance programs: A multi-method approach. Government Information Quarterly 2015;**32**(2):129-41.	No separate questions on social media.
Roberts LD, Rajah-Kanagasabai CJ. "I'd be so much more comfortable posting anonymously": Identified versus anonymous participation in student discussion boards. Australasian Journal of Educational Technology 2013;**29**(5):612-25.	Explores the impact of anonymity on student posting behaviour. No data on impact of researchers using data..
Robison C, Ruoti S, van der Horst T, et al. Private Facebook Chat. Los Alamitos, CA: IEEE Computer Society, 2012:451-60.	Study on personal privacy issues. Records percentage of respondents who think that chat conversations are private but in relation to personal privacy and identify theft etc.
Salmons J, Woodfield K. Social Media, Social Science & Research Ethics. London 2013	Survey of NSMNSS members on guidance and support for ethical conduct of research.
Scibelli DB. The trade-off: Consumer privacy for technology products and services. Dissertation Abstracts International Section A: Humanities and Social Sciences 2014;**74**(11-A(E))	Study aims to understand the level of personal information Internet technology users are willing to trade for perceived benefits of Internet based products and services.
Sharf BF. Beyond netiquette. The ethics of doing naturalistic discourse research on the internet. In: S J, editor. Doing internet research. London: Sage; 1999	Presents on lessons learnt from doing internet research. 13/14 posters on message board gave informed consent straight away for researcher to quote posts and the other poster gave permission after more detail on the research was offered.

Shibchurn J, Yan XB. Investigating Effects of Monetary Reward on Information Disclosure by Online Social Networks Users. 2014 47th Hawaii International Conference on System Sciences. 2014:1725-34.	Personal information to third parties. Related to age, gender etc. Not necessarily to researchers.
Shin D-H. The effects of trust, security and privacy in social networking: A security-based approach to understand the pattern of adoption. Interacting with Computers 2010;**22**(5):428-38.	Examines personal privacy, security and trust in social media sites only.
Shin W, Kang H. Adolescents' privacy concerns and information disclosure online: The role of parents and the Internet. Computers in Human Behavior 2016;**54**:114-23.	Looks at role of parents and the Internet in adolescents' online privacy concerns and information disclosing behaviors. Although respondents were asked are you concerned about the way certain websites or companies collect and use my personal information online. This related to personal information only.
Siegel DA. Will you accept the government's friend request? social networks and privacy concerns. PLoS ONE. 2013;8(11):	Personal privacy for security only.
Single ANV, Scott AM, Wale J. Developing Guidance on Ethics for Patient Groups Collecting and Reporting Patient Information for Health Technology Assessments. Patient 2016;9(1):1-4.	Discussion on guidance development.
Smit EG, Van Noort G, Voorveld HA. Understanding online behavioural advertising: User knowledge, privacy concerns and online coping behaviour in Europe. Computers in Human Behavior 2014;**32**:15-22.	This study investigates whether the existing knowledge is sufficient, whether website users are concerned about their privacy, and how they cope with OBA, cookies and the requested informed consent.
Sophos. 95% of Facebook users oppose privacy policy changes, Sophos poll reveals. Managing Information 2010;**17**(2):20-22.	Asked respondents "Do you think Facebook's proposed changes to their privacy policy are a good or bad thing". In March 2010, Facebook proposed changes to its privacy policy, paving the way for it to share personal information with "pre-approved" third-party websites. Personal information such as: name, profile picture, gender, friends and connections, userid, any content shared using the "Everyone" privacy setting.
Soczka L, Brites, Rui; Matos, Pedro. Personal information disclosure and perceptions about data usage by facebook. Proceedings of the 2nd European Conference on Social Media (ECSM 2015) 2015;():413-420.	Personal privacy. Self-disclosure related to perceptions of trust.
Spizman RJ, Miller MK. Plugged-in policing: Student perceptions of law enforcement's use of social media. Applied Psychology in Criminal Justice. 2013;9(2):100-23.	Personal privacy. Police investigating individual cases.
Steijn WMP, Vedder A. Privacy under Construction: A Developmental Perspective on Privacy Perception. Science Technology & Human Values 2015;**40**(4):615-37.	Study on personal privacy issues and data collection from government on individuals rather than research.
Stone MA. Sharing patient data : competing demands of privacy, trust and research in primary care. 2005.	Awareness and acceptance of data usage by researchers but not social media.

Suggate E. Layton D; Brown D; Shakir S. Patient-generated data and digital engagement-social media: Its impact and contribution to pharmacovigilance. Drug Safety 2015;38 (10):1031-1032.	Surveys professional's opinions on the value, quality, benefits and challenges of utilising patient adverse event reports (PAER) reports obtained via social media. **No mention of ethics but conference abstract obtained only.**
Swirsky ES, Hoop JG, Labott S. Using Social Media in Research: New Ethics for a New Meme? Am J Bioeth. 2014;14(10):60-1.	Editorial presenting two case studies.
Talib S, Ismail NA, Olowolayemo A, et al. Social networks privacy policy awareness among undergraduate students: The case of Twitter. 2014 5th International Conference on Information and Communication Technology for The Muslim World (ICT4M) 2014:5	Examines awareness of twitter sharing personal contact information to third parties but no further exploration.
Taylor H, Kuwana E, Wilfond BS. Ethical Implications of Social Media in Health Care Research. The American Journal of Bioethics. 2014;14(10):58-9.	Presents two case studies.
Thomas T. The Ethics of Social Media. Trial. 2014;50, 1:16-21.	Discussion paper
Till J. Research ethics: Internet-based research: Tripod; 2005 [cited 08/07/16]. Available from: http://ca916.tripod.com/index-3.html.	Discussion paper centered around three case studies.
Townsend L, Wallace C. Social Media Research: A Guide to Ethics. University of Aberdeen. ESRC. http://www.dotrural.ac.uk/socialmediaresearchethics.pdf	One objective is to gain user perceptions and expectations in regards to the use of their social media data for research. However, this is a guide to ethics without detail on methods and results for this objective.
Tu C-H. The measurement of social presence in an online learning environment. International Journal on E-Learning. 2002;1(2):34-45.	Personal privacy only
Tuunainen VK, Pitkanen O, Hovi M. Users' Awareness of Privacy on Online Social Networking sites - Case Facebook. 22nd Bled Econference-Eenablement: Facilitating an Open, Effective and Representative Esociety 2009:1-16.	Examines awareness of Facebook's privacy policy.
Uslaner EM. Trust, civic engagement, and the Internet. Political Communication. 2004;21(2):223-42.	Security and personal privacy only.
Van Royen K; Poels K; Vandebosch H. Harmonizing freedom and protection: Adolescents' voices on automatic monitoring of social networking sites. Children and Youth Services Review 2016;64:35-41	Monitoring of social media to detect harm such as cyberbullying and grooming.
Vayena E, Salathé M, Madoff LC, Brownstein JS. Ethical challenges of big data in public health. PLoS Comput Biol 2015;11:e1003904.	Discussion paper.
Wang Z, Yu Q. Privacy trust crisis of personal data in China in the era of Big Data: The survey and countermeasures. Computer Law & Security Review 2015;31(6):782-792	Trust with personal information by schools, government, companies etc.

Watson H, Finn RL. Ethical and Privacy Implications of the use of Social Media during the Eyjafjallajokull Eruption Crisis. International journal of information systems for crisis response and management. 2014;6, 4:29-41.	Personal privacy only
Wellcome Trust. Summary report of qualitative research into public attitudes to personal data and linking personal data. 2013.	General public's attitudes to different types of personal data and data linking.
Xie B, Watkins I, Golbeck J, Huang M. Understanding and Changing Older Adults' Perceptions and Learning of Social Media. Educational gerontology. 2012;38(4):282-96.	Personal privacy concerns only.
Yang HW, Liu H. Prior negative experience of online disclosure, privacy concerns, and regulatory support in Chinese social media. Chinese Journal of Communication 2014;**7**(1):40-59.	Study on disclosure of personal information for marketing but not research purposes.
Zimmer M. "But the data is already public" : on the ethics of research in Facebook. Ethics and Information Technology. 2010;12,4:313-25.	Uses a case study to discuss the ethics of research in Facebook
