# Supplementary material for: Attitudes Toward the Ethics of Research Using Social Media: A Systematic Review
Source: J Med Internet Res. 2017 Jun 6;19(6):e195. doi: 10.2196/jmir.7082 (PMC5478799; doi:10.2196/jmir.7082)
Supplement: Multimedia Appendix 4 [file jmir_v19i6e195_app4.pdf]

Appendix 4: Characteristics of included studies

Source	Research Question	Number of respondents (including response rate)	Characteristics of the participants	Sampling methods/survey distribution	Specific questions or methods used to ascertain ethical considerations	Key findings identified in terms of the ethical considerations raised
Researchers as respondents						
Alim 2014 [43]	To gain researchers experiences of, and thoughts about, ethical research practices associated with automated extraction.	Sent to 400 researchers. 84 responses 20 invalid as only section one completed leaving behind 64 responses.	Researchers and academics. 31% female, 69% male, Mean age: 29. 59% computer scientists, 15% social scientists, 7% engineering/physical sciences, 5% other, 4% arts and humanities, 3% biological sciences, 3% business and management. 42% PhD students, 34% academic, 15% post-doctoral researchers/research assistant, 5% independent researcher, 4% other. 36% Europe, 30% North America, 23% Asia, 3% Australia, 3% South America, 5% unknown.	Respondents identified by searching academic papers in Google Scholar. First authors emailed the questionnaire link and encouraged to pass the questionnaire onto interested researchers or academics (snowball sampling). Alim also emailed questionnaire to own academic contacts. Questionnaire link was emailed to social network university research groups and placed on a social network analysis mailing list.	Open and closed questions to address;<br>1: What are the most popular ethical considerations implemented in research studies involving automated extraction from user profiles?<br>2: What are the reasons for the lack of implementation of less popular ethical considerations in research studies involving automated extraction from user profiles?<br>3: What ethical challenges are faced by researchers carrying out research studies in this area?<br>4: What are the issues and concerns that researchers have for the future with regard to ethical research practices?	Researchers becoming more aware of ethical considerations. However, require more clarity in areas such as informed consent and public and private data. More research required on user's ethical needs.

Bakardjieva 2001 [56]	To examine the views of subjects in a virtual community regarding their posts being analysed for research purposes and the ethical challenges faced by researchers in the process.	Not known.	Mailing list discussants. No further details presented.	Received, read and kept the discussions provoked by post on a mailing list.	Moderator posted call by researchers for participation on mailing list. Researchers then recorded feelings and concerns of potential research subjects in subsequent discussions on mailing list.	An ethical approach to online research is practically achievable through a process of preliminary engagement with the group and/or involvement of group members in the planning and designing of the study.
Carter 2015 [68]	To understand how academics tasked with ethically reviewing research proposals perceive the ethical challenges posed by social media research? And how do attitudes towards social media research ethics (SMRE) relate to experience of reviewing research proposals of this type, and experience of Internet-mediated ethical guidelines	30 academic members of staff	Academic staff from single UK university, 18 males, 10 females, 2 undeclared. Median age 35, modal age 44. All five faculties at the institution were represented in the sample (science, 9 respondents, social sciences – 5, and medicine and health sciences – 5).	Emailed request containing a hyperlink to an online survey, sent via the respective Heads of the institution's 26 School Ethics Committees.	Respondents asked to agree, disagree or neither to 12 statements. 1 "No need for informed consent if social media data publicly accessible" 2 "Informed consent required to enable withdrawal from social media research", 3 "Unlikely that individuals will be identified if social media dataset is anonymised", 4 "Informed consent creates more problems for social media researchers than necessary", 5 "Too impractical to apply all ethical considerations to social media research", 6	Many of the responses present a complex picture in which respondents recognise the importance of avoiding deception and gaining consent, but also acknowledge the problems in doing so. Most respondents disagreed that studying public social media data was essentially the same as studying documented text and that individuals wouldn't be identified from large datasets if anonymous yet

	and training?				“Responsibility is upon individuals if they do not wish to participate in social media research”, 7 “Acceptable to use public social media data without informed consent”, 8 “No ethical difference between studying offline and social media behaviour in public spaces”, 9 “Benefits of studying behaviour on social media outweigh need for informed consent”, 10 “Studying public data on social media data is essentially the same as studying documented text”, 11 “User agreement with social media ‘terms and conditions’ sufficient as informed consent”, 12 “Acceptable to deceive social media users in research as long as informed at a later date”	levels of agreement and disagreement were roughly equivocal with respect to the acceptability of using such data without informed consent, the ethical equivalence of researching in offline and online public spaces, and the responsibility of users in indicating willingness to participate.
Denecke 2014 [69]	To summarise the current awareness of the research community with respect to ethical issues to be considered within public health monitoring and	45 researchers from 3 research groups replied	Registered members of mailing list. No details given.	Questions asked via the International Medical Informatics Association (IMIA) Social Media Working Group mailing list (with 45 registered members)	Asked 3 questions in relation to public health monitoring. The first question related to privacy – “Is privacy of individuals violated when social media data is exploited a) by health organisations for public	Different social media platforms should be managed in different ways in terms of confidentiality and privacy. People should be aware that an open

	research using medical social media data.				health monitoring, b) by researcher?" The other 2 questions related to the responsibilities of health organisations and researchers when problems are identified on social media and the value of social media.	platform is accessible to all. For a closed group the administrator should be asked to gain access. Individuals should be de-identified and cited only indirectly.
McKee 2009 [70]	To present ethical issues of internet research faced by researchers and how they thought through these issues.	30 Internet researchers	Researchers from corporate research centres and academia in 11 countries -Australia, Canada, India, Italy, Japan, Korea, New Zealand, Norway, United Kingdom, Taiwan and United States, working in variety of disciplines including anthropology, communication, economics, education, gaming, gender, information technology, media, psychology, rhetoric and writing, sociology and technical communication. Primarily qualitative researchers but also several qualitative researchers. Graduate students to new and established professors.	Contacted researchers known personally or known through published work and/or conference presentations and contacted researchers based on recommendations from researchers interviewed.	Used open-ended interviews on which to base book.	Researchers strived to follow 'do no harm' principle. Common agreement that there cannot be blanket approach to internet research ethics– contextual details matter. Each research situation is unique. Thus ethical decision-making can only proceed on an individual case by case basis.
Woodfield 2013 [71]/Salmons	To explore the impact of social	465 registered members of New	International network of interdisciplinary	Programme of on and offline activities	Throughout the activities the topic of	Discussions focused 1)

2013 [42]	media on social science research including how social media research changes researchers perceptions of ethical practice.	Social Media, New Social Science? Network (NSMNSS)	researchers (representing over 20 academic disciplines). 59% described their level of experience as 'expert' or 'intermediate'75% based in HEIs, 25% in applied research. 65% are UK based, remaining 35% from Europe, North America, Asia and Pacific Rim.	including full-day conference and hour long Twitter chat around themed topics.	ethics in social media research was consistently raised.	informed consent, 2) confidentiality /anonymity, 3) role and safety of the researcher, and 4) research setting or social media platform. There was concern of a lack of agreement on ethical frameworks for social media research. Yet for some this gave freedom to the methods and approaches they adopt.
Social media users as respondents						
Beninger 2014 [57]	To understand what social media users think constitutes 'good' ethical practice in online and social media research.	34 people (4 focus groups and 2 paired interviews and 2 one-on-one depth interviews)	18 male, 16 female. Age 18-25 (7 respondents), 26-35 (8), 36-49 (8), 50-60 (5), 61+ (6). Low users of social media (10 respondents), medium (12) and high (12).	Sample of participants from British Social Attitudes 29 (BSA 29) survey who agreed to be re-contacted and external recruitment agency to supplement the sample.	Vignettes used to illustrate key points and simulate discussion to explore the ethical considerations from a user's perspectives for researchers using social media research	
Bond 2013 [57]	To explore the views of contributors to discussion boards with regards to if (and how) they feel their contributions to boards should be used by health	33 individuals expressed an interest and 30 consented. 4 did not complete the interview questions leaving 26 respondents.	12 male, 9 female, 6 unclassified (identified from username/email correspondence). All participants were sufficiently fluent in written English and had made at least one post to one of the 4 diabetes forums selected.	Requests for participation posted on 4 diabetes forums. None of these forums required membership to read posts and 2 required membership to post.	Online semi-structured asynchronous (email) interviews in which participants were sent several emails. Each email contained 1-2 questions to ascertain whether contributors to online diabetes discussion boards felt it	Participants agreed that forum posts are in the public domain and that aggregated information could be freely used by researchers.

	researchers.				was acceptable for researchers to use information on health discussion boards, what permission should be sought prior to using this information, and whether the length of time since the post was made influenced the need to obtain permission.	
Chen 2004 [58]	To provide an understanding of the view of online participants regarding the appropriate conduct for online research by information gatherers (academic researchers and journalists).	47 respondents	9 mailing list owners and 38 moderators/long standing members of newsgroups. Lists or newsgroups dealt with sensitive and controversial topics (such as depression and abortion).	Selected 34 mailing lists and 94 newsgroups on sensitive and controversial topics. Emailed survey questionnaire to 32 list owners. Sent out calls on 94 newsgroups for moderators or long standing members, 110 individuals responded to the call and the authors then sent out questionnaires to the moderators and long standing members identified.	Asked to describe their experiences of dealing with researchers and journalists, their reservations concerning these people using their lists or group for research, as well as their recommendations for such research activity.	There was general animosity towards researchers. Any research undertaken should be conditional based on research identify disclosure, informed consent and feedback.
Evans 2015 [59]	To gain stakeholders and social media users views on how the research industry should use social media in an ethical way.	1250 respondents to online qualitative survey, 3 qualitative workshops and 9+ interviews with experts.	1250 adults aged 16-75 (survey), 2 workshops with adults and one with 13-15 year olds. Interviews with experts from 9 organisations. States workshop participants recruited to be broadly reflective of	No information provided about how the subjects were recruited for online survey, workshops or interviews.	Full survey is presented in appendix – aims to ascertain people's attitudes towards possible uses of their social media data and the value of social media research. Survey included questions on	60% of social media users do not support the use of social media data for research. Generally felt that terms and conditions shouldn't be

			UK population.		which types of use of social media data should not happen and conjoint analysis on data asking if respondents would approve series of example research projects on a scale of 1 to 10.	sufficient for informed consent and people need to be given the option to opt out. Biggest factor in the likelihood to approve research is whether the data is already publically available.
Hudson 2004 [60]and Hudson 2005 [61]	To record how potential subjects respond to being studied in chatrooms.	2260 unique usernames (there is not necessarily a one-to-one mapping between individuals and usernames).	Chatrooms on ICQ chat (with moderators and active conversations) purposefully selected to represent range of sizes and avoiding sensitive discussion such as ‘breast cancer survivors’. Wide range of topics including geographical region or language, age-orientated, romance or friends, adult or sexuality, technical, trivia and miscellaneous.	Randomly assigned 137 chatrooms into 4 groups. In group 1 posted a message to tell participants being recorded, in group 2 posted an opt-in message, and group 3 posted an opt-out message. In group 4 did not post a message but simply entered using nickname “Chat_Study”.	Recorded if ‘kicked-out’ of chatroom within 5 minutes. Examined any messages explaining why kicked out and comments directly pertaining to the researchers.	Kicked out of 63.3% of the chatrooms in groups 1 to 3 compared with 29% of the chatrooms in group 4. No significant differences between groups 1 to 3. When given a chance to opt in, only 4 of 766 potential subjects did so and when given chance to opt out 2 of 443 did so. Reasons for being kicked out were prohibition of spamming, opposition to being studied, general requests to leave and insults.
Michaelidou 2016a [62] and	To develop a quantitative	28 participants in 4 focus groups and	Focus groups: professionals who were	Not reported.	Focus groups analysed using template analysis	10-item scale for measuring

Michaelidou 2016b [63]	instrument that captures consumers' ethical perceptions regarding how they are researched on social media	377 respondents to online survey	social media users, aged 18 and over.<br>Survey respondents: Pilot sample of 107 - 55% male and 46% female, age 18-25, 21%; 26-35, 23%; and 36-45, 18%, and further education, 23%; undergraduate degree, 30%; and postgraduate degree, 25%.<br>No detail on second sample of online survey respondents.		and coded using QSR NVivo. 54 items in pool. Online survey analysed using exploratory factor analysis (EFA) and confirmatory factor analysis (CFA).The aim was to measure the unidimensionality of each emerging factor.	consumers' ethical perceptions of social media research. 10 factors were transparency, legality, approval, privacy concerns, permission, vulnerability, reward, consumer responsibility, protection (by companies and governments), and terms.
Mikal 2016 [64]	To investigate public attitudes towards using Twitter for population-level depression monitoring.	26 participants in 5 focus group interviews	17 male, 8 female. Average age 26.9. 16 reported depression history and 10 reported no depression history. Various professions: computer/ technology (5 respondents), office/administrative (8), education (1), students (7), specialized services (4), and stay-at-home parent (1). Twitter use ranged from passive/content receiving to active/content generating.	Advertised on list serves, Internet discussion boards and flyers on local internet community websites.	Semi-structured interviews around 5 themes: (1) Twitter use (2) privacy expectations, (3) attitudes towards population level mental health monitoring, (4) individual versus aggregate health monitoring, and (5) views on regulating social media mining.	Relatively positive view provided the data are anonymous and aggregated to protect identities.
Monks 2015 [65]	To determine how young people perceive the feasibility and acceptability of	48 students	8 focus groups (45 minutes log) with 6 13-14 years old Western Australian school students per group.	Sample of convenience drawn from students participating in a leadership workshop.	3 main questions. 1) How do young people perceive the appropriateness of different methods of	Young people expressed some concerns about their privacy but were open to the

	social media as a platform for conducting research with and/or about them in relation to various mental health and wellbeing issues.				online research compared to offline research? 2) What factors would influence young people's participation in research via different forms of social media? and 3) What are young people's perceptions of the issues of consent and privacy around the use of their social media data for research purposes?	use of social media for research if they were given an opportunity to provide consent and assured of confidentiality and anonymity.
Moreno 2012 [38]	To determine older adolescents' responses after learning that they were participants in a research study that involved identification of participants using Facebook.	132 participants	Older adolescents aged 18-19; average age was 18.4 years (SD=.5); 64 male, 68 female; 120 white. 5 asian, 4 hispanic, 1 african American and 1 mixed race.	Used Facebook search engine to identify profiles of freshmen undergraduate students within one large state university in U.S.. Search yielded 416 profiles. After initial exclusions (e.g. age not 18 or 19; no contact info) 188 profiles were eligible. For profiles that met criteria, owners invited by phone to participate in one to one interview. $50 incentive for those that completed interview.	At the end of interview about health measures participants were asked "We identified potential participants for this study by looking at publicly available Facebook profiles of people in the university network. Do you have any thoughts about that?"	Endorsement was given by 26 respondents. 48 expressed that they were fine with the experience 38 were neutral or had no specific comments. 12 were uneasy, 8 fit had overt concerns.
Petersen 2013 [66]	To assess attitudes of health discussion group participants regarding	27 respondents	Survey response rates were 2% (11 of 539) for the Medical Webmasters (MWM) list and 10.4% (16 of 154) for the	Survey posted to two electronic lists - Medical Webmasters (MWM) an open, unmoderated list for individuals operating	Questions on whether journalists; may join lists to research stories, quote list subscribers comments in	Two themes emerged. Respondents believed that journalists should

	journalists' access to and use of list and archived postings.<br><br>NB Authors state "Because survey respondents view journalistic endeavors as research, they are likely to hold the same expectation of researchers."		Patient Advocates in Research (PAIR) list.	medical and health-related web sites;, and Patient Advocates in Research (PAIR) a closed, unmoderated list for cancer patient advocates. Responses were accepted for seven days.	publications, and to quote list subscribers without informing the list of the publication venue(s) and date(s).	seek permission from list members and/or webmasters and viewed members desire for privacy as taking precedence over researchers' goals.
Williams 2015 [67]	To ascertain users' perceptions of the use of their social media posts.	564 respondents	Social media users	Online survey	Survey had open questions to capture qualitative responses and included sections on; awareness of Terms of Service and attitudinal questions on informed consent, anonymization and type of researcher (commercial, government, police & university).	37% are not at all concerned about their social media information being used by university researchers, whereas 46% are slightly concerned, 11% are quite concerned and 5% are very concerned.
