# Supplementary material for: Attitudes Toward the Ethics of Research Using Social Media: A Systematic Review
Source: J Med Internet Res. 2017 Jun 6;19(6):e195. doi: 10.2196/jmir.7082 (PMC5478799; doi:10.2196/jmir.7082)
Supplement: Multimedia Appendix 5 [file jmir_v19i6e195_app5.pdf]

Appendix 5: Quality of reporting in included studies

Source	Question and study design	Selection of participants	Method of data collection	Methods of data analysis
**Researchers as respondents**				
Alim 2014 [43]	Yes	Yes	Yes	No
Bakardjieva 2001 [56]	Yes	No	No	No
Carter 2015 [68]	Yes	Yes	Yes	Yes
Denecke 2014 [69]	Yes	Yes	No	No
McKee 2009 [70]	Yes	Yes	No	No
Woodfield 2013 [71] /Salmons 2013 [42]	No	No	No	No
**Social media users as respondents**				
Bond 2013 [40]	Yes	Yes	Yes	Yes
Beninger 2014 [57]	Yes	Yes	Yes	No
Chen 2004 [58]	Yes	Yes	No	No
Evans 2015 [59]	Yes	No	Yes	No
Hudson 2004 [60] / 2005 [61]	Yes	Yes	No	No
Michaelidou 2016a [62] /2016b [63]	Yes	No	No	Yes
Mikal 2016 [64]	Yes	Yes	Yes	Yes
Monks 2015 [65]	Yes	Yes	Yes	Yes
Moreno 2012 [38]	Yes	Yes	Yes	Yes
Peterson 2013 [66]	Yes	Yes	Yes	No
Williams 2015 [67]	No	No	Yes	No
