# Supplementary material for: Attitudes Toward the Ethics of Research Using Social Media: A Systematic Review
Source: J Med Internet Res. 2017 Jun 6;19(6):e195. doi: 10.2196/jmir.7082 (PMC5478799; doi:10.2196/jmir.7082)
Supplement: Multimedia Appendix 6 [file jmir_v19i6e195_app6.pdf]

	Researchers				Social media users				Consent		Social media site responsibilities			
**Source**	**Perceived benefit of research**	**Type of researcher**	**Validity of the research methods**	**Risks to researchers**	**Risks to social media users**	**Vulnerable groups**	**Original purpose of posts**	**Privacy and self-regulation**	**Informed consent**	**Researcher disclosure**	**Terms of service**	**Site administrators**	**Legalit y**	**Type of platform**
**Researchers as respondents**														
Alim 2014				✓	✓	✓		✓	✓		✓		✓	
Bakardjieva 2001	✓				✓		✓	✓	✓	✓			✓	
Carter 2015								✓	✓	✓	✓			
Denecke 2014		✓						✓	✓		✓	✓	✓	✓
McKee 2009				✓	✓	✓	✓	✓	✓	✓			✓	
Woodfield 2013/Salmons 2013		✓		✓		✓	✓	✓		✓	✓			✓
**Social media users as respondents**														
Beninger 2014	✓	✓	✓		✓	✓	✓	✓	✓	✓	✓		✓	✓
Bond 2013	✓						✓	✓	✓	✓		✓	✓	✓
Chen 2004	✓	✓	✓	✓	✓	✓		✓	✓	✓		✓	✓	✓
Evans 2015	✓	✓				✓		✓		✓	✓		✓	
Hudson 2004/2005				✓	✓	✓				✓				
Michaelidou 2016a/2016b	✓					✓		✓	✓				✓	
Mikal 2016	✓	✓	✓		✓	✓		✓	✓	✓	✓		✓	✓
Monks 2015	✓	✓	✓		✓	✓	✓	✓	✓	✓	✓			✓
Moreno 2012	✓				✓			✓	✓					
Petersen 2013	✓				✓	✓		✓	✓	✓		✓	✓	
Williams 2015		✓						✓	✓		✓			

Appendix 6: Results matrix for studies by emerging themes
